# Technical challenges in establishing local native T1 reference ranges in cardiac MRI: a critical review

**DOI:** 10.1186/s13244-026-02370-w

**Published:** 2026-08-24

**Authors:** Maheran Che Ha, Khairil Amir Sayuti, Yusri Mohammed, Mardhiyati Mohd Yunus, Nurisya Mohd Shah, Mohd Mustafa Awang Kechik, Mohammad Johari Ibahim, Muhammad Khalis Abdul Karim

**Affiliations:** 1 Department of Physics, Faculty of Science, Universiti Putra Malaysia (UPM), Serdang, Malaysia; 2 Department of Radiology, Hospital Universiti Sains Malaysia, Kubang Kerian, Malaysia; 3 Department of Radiology, Hospital Sultan Idris Shah, Serdang, Malaysia; 4 Faculty of Health Science, Universiti Selangor (UNISEL), Shah Alam, Malaysia; 5 Faculty of Medicine, Universiti Teknologi Mara (UiTM), Jalan Hospital, Sungai Buloh, Malaysia; 6 Institute for Mathematical Research (INSPEM), Universiti Putra Malaysia (UPM), Serdang, Malaysia

**Keywords:** Magnetic resonance imaging, Myocardium, Reference value, Reproducibility of results, Quality control

## Abstract

**Abstract:**

Establishing local reference ranges for native T1 in healthy myocardium is critical in cardiac magnetic resonance imaging (CMR) for the accurate detection of myocardial fibrosis and other tissue abnormalities. However, native T1 measurements vary widely and are sensitive to technical factors such as the magnetic field strength, static magnetic field (B0) and radiofrequency (B1) homogeneities, differences in MRI scanners across various vendors, imaging protocols, and specific acquisition parameters employed. These variabilities have consequently hindered the direct comparison of T1 values across different studies and centers. This review aims to evaluate the main technical challenges and methodological inconsistencies that impact the establishment of local native T1 reference ranges in CMR, and review proposals for mitigation, harmonization, and standardization strategies designed to enhance comparability across CMR platforms. A comprehensive literature search was conducted focusing on myocardial native T1 mapping using the modified Look-Locker inversion recovery (MOLLI) technique and its variants, as well as on alternative T1 mapping sequences like ShMOLLI, SASHA, SMART1Map, and SAPPHIRE. Reported native T1 values in healthy myocardium were found to be inconsistent across studies, driven by differences in the magnetic field strength, static magnetic field (B0), and radiofrequency (B1) homogeneities, and vendor-specific hardware and software configurations. Standardization of T1 mapping protocols, including standardized imaging parameters and vendor-specific reference adjustment, is therefore necessary to improve the comparability and reliability of myocardial T1 values across CMR centers. In addition, a robust quality assurance (QA) program and effective quality control (QC) measures are pivotal in ensuring the reliability and consistency of the measurements.

**Key Points:**

- **Question** How do technical factors and scanner-specific variabilities hinder the establishment of reliable local native T1 reference ranges for accurate myocardial tissue characterization?
- **Findings** Standardization of acquisition parameters, vendor-specific benchmarks, and robust quality assurance programs effectively minimize inter-scanner variability, ensuring reliable and comparable native myocardial T1 measurements across centers.
- **Critical relevance statement** This review identifies technical drivers of T1 variability and proposes a standardization framework, enabling clinicians to establish accurate local reference ranges and improve diagnostic comparability across diverse platforms in clinical radiology practice.

**Graphical Abstract:**

**Figure:**
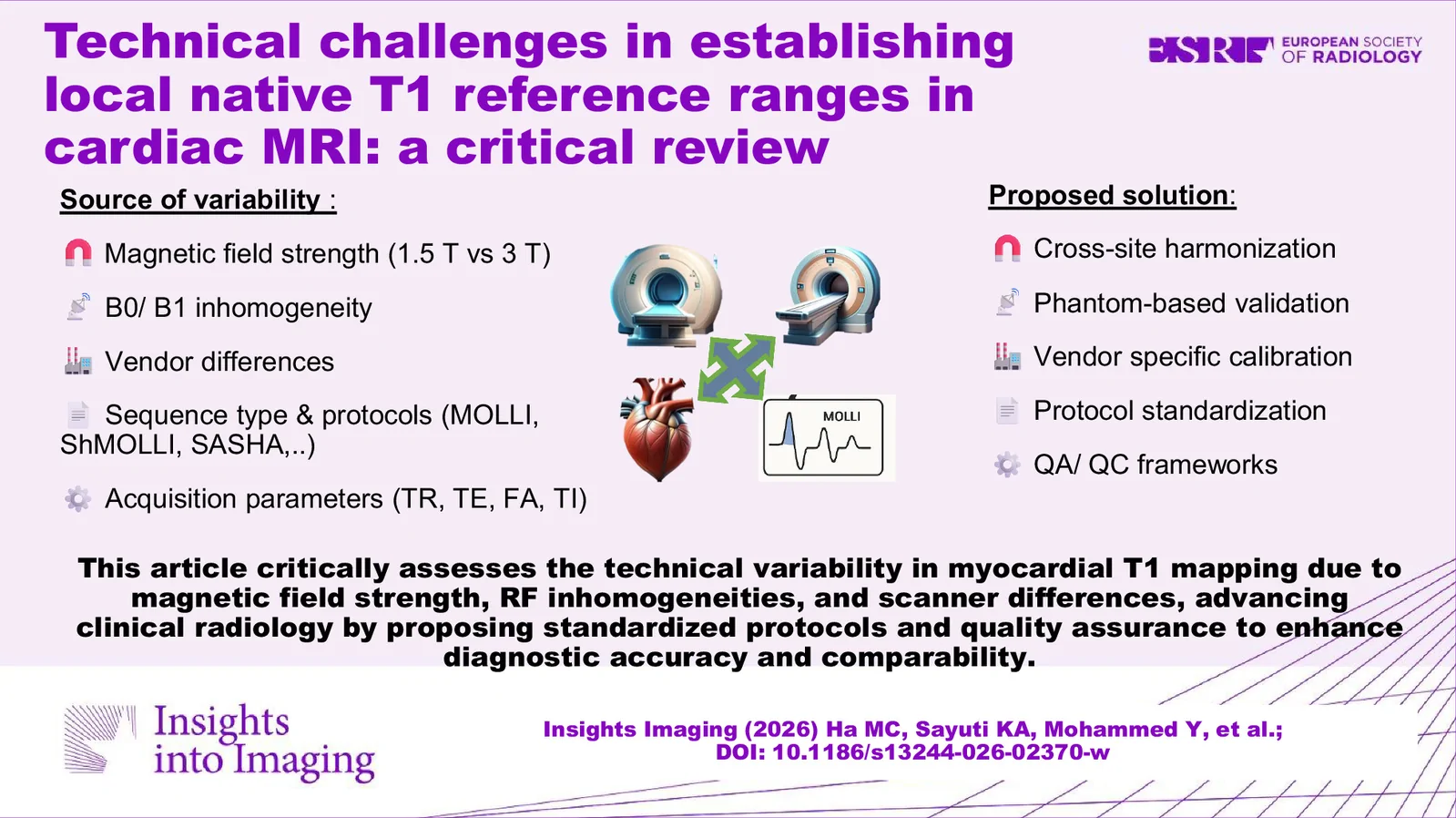

## Introduction

Cardiac magnetic resonance imaging (CMR) is a non-invasive imaging modality that enables the comprehensive evaluation of the heart structure and function, as well as tissue composition [1]. CMR protocols typically include multiple pulse sequences that provide different types of images. Among them, T1 mapping has emerged as a promising tool for characterizing myocardial tissue [2, 3]. This is a quantitative technique used to quantify the longitudinal relaxation time (T1), which is an intrinsic tissue property that reflects how quickly the longitudinal magnetization recovers after a radiofrequency (RF) excitation [4]. The T1 value refers to the time constant when the tissue of interest achieves around 63% of its longitudinal relaxation. The value measured from non-contrast images is known as the native T1 value. It provides absolute and objective quantitative values [5], enabling the detection of subtle changes that are an important indicator for pathology assessment (e.g., cardiac pathology) [6].

Direct measurement of myocardial T1 in the beating heart was historically challenging, but the development of the MOLLI sequence in 2004 made routine cardiac T1 mapping feasible [7]. MOLLI allows a series of images at different inversion times (TI) to be acquired within a single breath-hold without compromising image quality. Research on cardiac T1 mapping has since been actively pursued, resulting in significant clinical applications in cardiac T1 mapping. For instance, native T1 mapping can detect diffuse fibrosis and myocarditis, aid in edema assessment in acute myocardial infarction, and improve the diagnosis of various cardiomyopathies [8–10]. Furthermore, it also has prognostic value in predicting adverse cardiac events based on the extent of tissue abnormalities and the increment in native T1 value [11–13]. Despite its significant potential, measured T1 values are sensitive to both technical and biological factors [14, 15].

In 2017, the Society for Cardiovascular Magnetic Resonance (SCMR) recognized this variability and issued a consensus statement advising each center intending to conduct and officially document quantitative T1 mapping to establish their own local reference range [16]. Centers are advised to benchmark their T1 values against published reference values obtained under similar conditions. The goal is to ensure accuracy and reliability in clinical interpretation by accounting for any local scanner or protocol biases.

Despite this guideline, a comprehensive understanding of the technical influence on T1 value remains lacking. Prior reviews of T1 mapping have focused on its clinical applications [17–19]. Few have specifically zeroed in on the specific challenges in establishing normal T1 ranges for healthy myocardium across different settings. Therefore, this review aims to focus on the technical factors that drive variability in myocardial T1 values and to highlight previously proposed solutions to these challenges. By concentrating on these issues, we aim to provide guidance for researchers and clinicians working to establish reliable local T1 reference ranges and to facilitate harmonization of T1 mapping across centers.

## MRI technology and technical factors affecting T1 mapping

The accuracy and consistency of T1 measurement can be influenced by a wide array of MRI system-related factors, particularly the magnetic field strength and the uniformity of both the static magnetic field (B0) and the radiofrequency transmit field (B1). This section outlines how these factors contribute to variation in T1 values and highlights considerations for minimizing their impact in clinical and research settings.

### Main magnetic field strength (B0)

In MRI, a strong and uniform static magnetic field (B0) aligns hydrogen protons in the body to generate measurable signals. Higher field strength widens the energy gap between proton spin states, thus hindering their relaxation to equilibrium. Consequently, T1 relaxation time increases with increasing field strength, making T1 values inherently field dependent [20, 21].

Although modern high-field MRI scanners (e.g., 1.5 T, 3 T) implement automated shimming to achieve B0 uniformity, residual inhomogeneity remains, especially in complex anatomical regions such as the thorax. These B0 variations introduce spatially dependent off-resonance effects, leading to a deviation in local precessional frequencies that can alter the magnetization evolution during T1 mapping sequences. As a result, even small B0 inhomogeneities can contribute to regional variability in T1 measurements, thereby reducing the reproducibility and accuracy of native T1 across imaging sites and scanner platforms [22]. This effect is worse at 3 T compared to 1.5 T, where the spread of reported native T1 values in healthy myocardium is broader [23]. Crucially, the variation in T1 values due to these effects reflects differences in physics rather than true tissue properties [24].

**Fig. 1:**
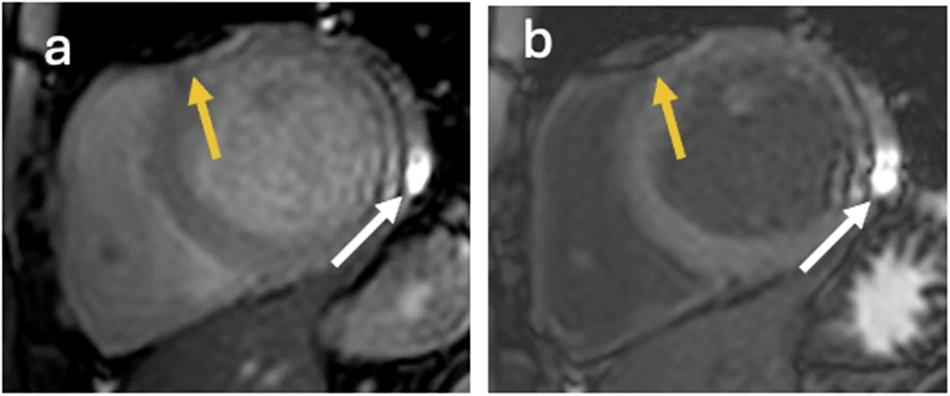
Susceptibility artifact in the inferolateral wall (white arrows), and off-resonance related dark banding artifact (yellow arrows) in the anterior wall can be seen in both **a** native T1 mapping and **b** post-contrast T1 mapping

Furthermore, the balanced steady-state free precession (bSSFP) sequence, which is typically used as the readout in MOLLI (T1 mapping, e.g., 5(3s)3 scheme) for cardiac imaging, is inherently sensitive to the off-resonance effect caused by B0 inhomogeneity [25]. The frequency shift in a non-uniform B0 field leads to periodic modulation of the steady-state signal, producing signal voids and banding artifacts, particularly in areas near the lung and heart where susceptibility differences are high (as shown in Fig. 1) [22, 26]. The off-resonance variation across the heart had been measured in 1.5 T and 3 T, where it was found to vary by approximately 62 Hz and 125 Hz, leading to T1 variation of 20 ms and > 50 ms, respectively [25], and resulting in artifactual regional variation in T1 values.

Given the substantial influence of B0 inhomogeneity on the accuracy and reproducibility of T1 values, particularly in sequences like bSSFP, various mitigation strategies have been proposed to minimize off-resonance effects and enhance field uniformity. One earlier proposed strategy is simply to replace the bSSFP readout with a spoiled gradient echo (GRE) readout, which is known to be more robust against B0 inhomogeneity [27]. However, images from bSSFP readout generally offer higher quality compared to other gradient echo sequences, making it the sequence of choice [20]. Therefore, implementing optimal acquisition parameters based on a comprehensive understanding of the basic principle of bSSFP will help mitigate this effect [28] (refer to the “Acquisition parameters and optimization” section). Regarding anatomical influence, T1 measurement is suggested to be performed at the septal region (refer to the “Slice selection, myocardial segmentation, and ROI placement” section). Additionally, with current technological advancement, lower field strength (e.g., 0.55 T) has been proposed as an alternative for cardiac imaging, specifically to reduce this effect [29].

**Fig. 2:**
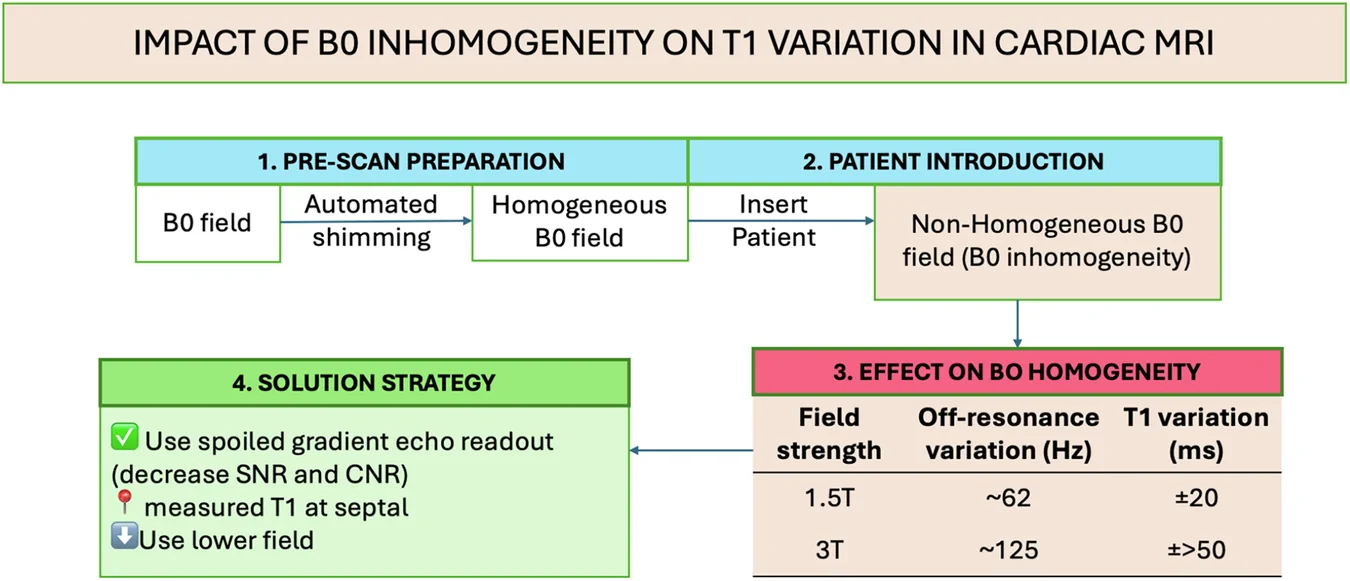
Impact of B0 inhomogeneity on T1 variation in cardiac MRI

In summary (Fig. 2), as a sequence-independent factor and system-level factor that universally affects native T1 values, increasing B0 will simultaneously increase T1 values due to the increment in the energy gap, which slows the longitudinal relaxation. This effect is independent of the pulse sequence or vendor implementation. This highlights the need for site-specific T1 benchmarks that account for field strength when comparing clinical results across institutions.

### Transmit RF field homogeneity (B1)

Transmit RF field (B1) homogeneity refers to the uniformity of the RF field transmitted by the MRI system to excite protons in the body. It delivers the desired flip angle (FA) during pulse sequences, which directly influences the accuracy of tissue signal and contrast. In an ideal scenario, the B1 field would be spatially uniform across the imaging volume, ensuring consistent excitation. However, in practice, B1 inhomogeneity or a non-uniform B1 field commonly occurs due to factors such as coil design, dielectric effects, patient-specific anatomy, and the use of higher field strength (e.g., 3 T).

**Fig. 3:**
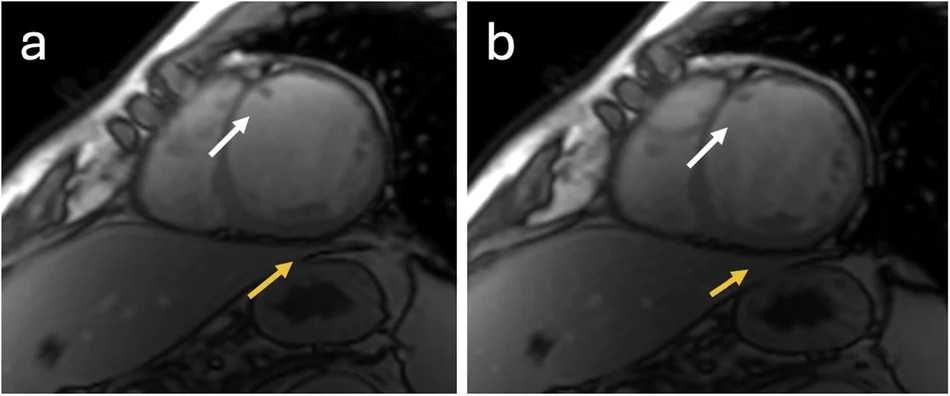
Inhomogenous signal intensity due to B1 inhomogeneity can be observed qualitatively by looking at the differences in signal intensity. The anterior region notably has higher signal intensity (white arrows), as compared to the inferior region (yellow arrows). Both images are taken from the 3-T MRI of the same patient but at different RR intervals: **a** 1050 ms and **b** 955 ms

This inhomogeneity leads to regional variations in the achieved FA [30, 31], resulting in variations in signal intensity (Fig. 3), introducing errors in signal quantification, and consequently affecting the accuracy and reproducibility of T1 mapping. This means that if the effective FA is set to 35°, some regions might receive 30°, while others get 40°, even though the system intends for all areas to receive 35°. At 3 T, the wavelength is on the order of the chest dimensions, which causes more pronounced non-uniformity as compared to 1.5 T. This is supported by a study that performed T1 measurement on both systems and found that regional variation in T1 value across myocardial segments was more significant on 3 T as compared to 1.5 T [32].

In combating this issue, a multi-channel transmit coil can be used to create a more uniform B1 field across the imaging volume, in addition to the use of B1 shimming [33]. Additionally, dual source parallel excitation can also further improve B1 inhomogeneity, especially on 3-T systems [34]. Furthermore, parallel transmission with multiple independent channels can be employed for even finer control [35]. Moreover, transient phase B1 mapping is another approach for bSSFP-based imaging, which has shown promising results in compensating for the inhomogeneity [30]. Using lower flip angles on a 3-T system can mitigate the impact of B1 variations, leading to improved B1 homogeneity at the cost of reduced signal-to-noise ratio (SNR) [25].

**Fig. 4:**
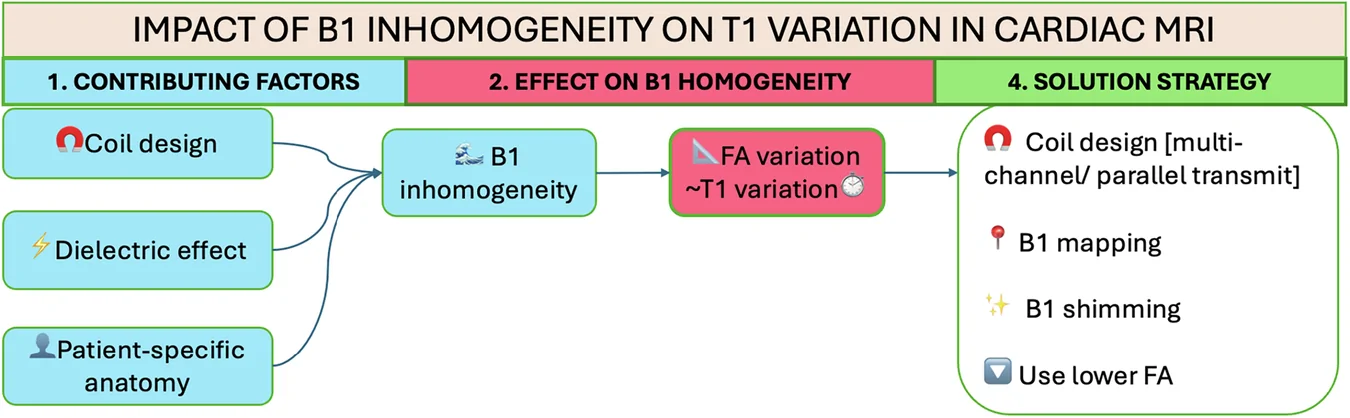
Impact of B1 inhomogeneities on T1 variation in cardiac MRI

In summary (Fig. 4), B1 inhomogeneity is a significant source of variability in T1 mapping as it affects FA accuracy across all sequences and vendors, especially on 3-T systems where dielectric effects are more pronounced [36]. Without proper correction or calibration, such variations may impact clinical thresholds for detecting subtle myocardial abnormalities. Improving coil design to increase efficiency is one of the mitigation options that has been a subject of active research. While waiting for a robust coil design, the simple solution of lowering the FA can help in improving B1 uniformity.

### Manufacturer and scanner model variability

**Fig. 5:**
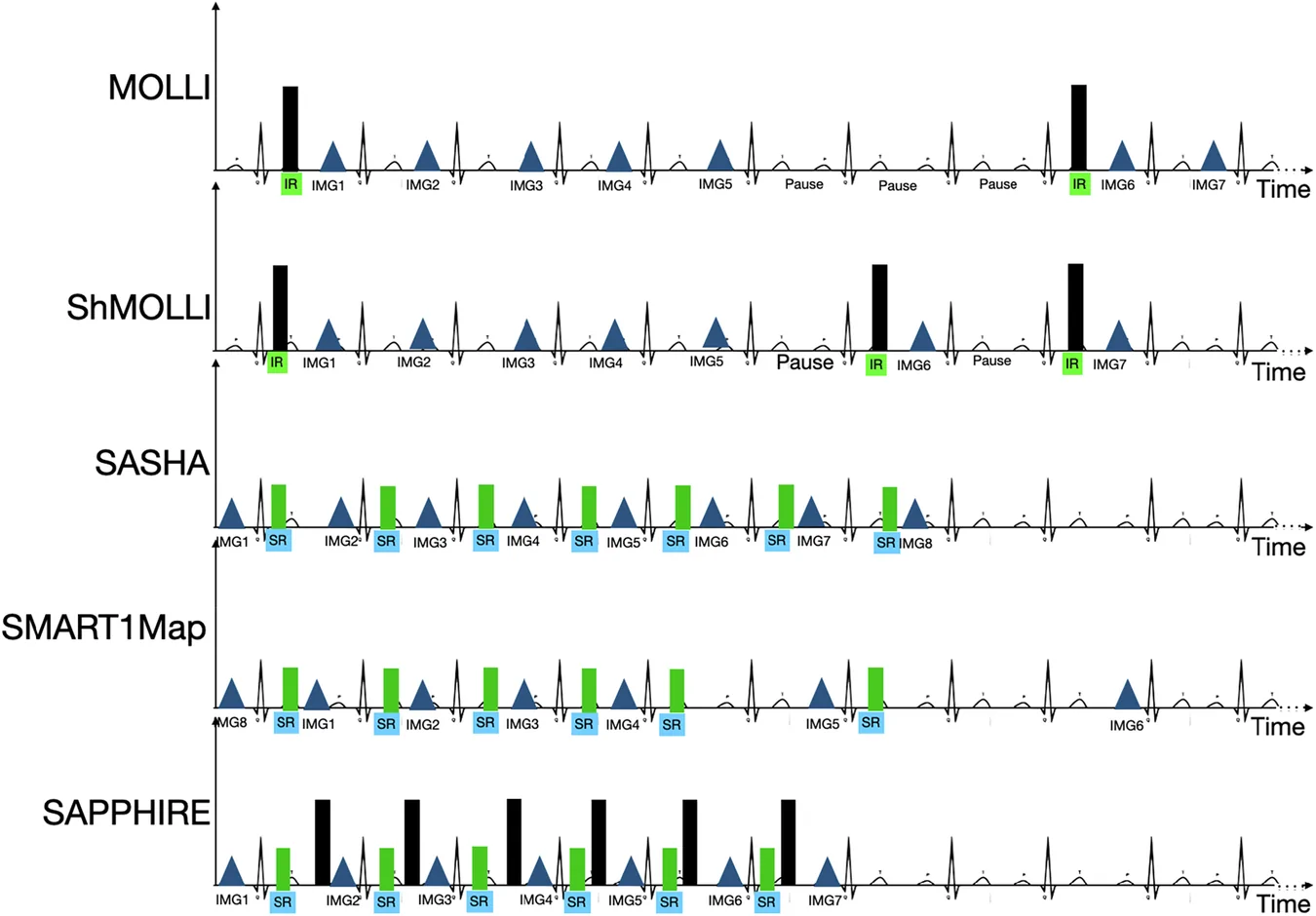
Schematic diagram of pulse sequence design for different T1 mapping techniques: MOLLI, ShMOLLI, SASHA, SMART1Map, and SAPPHIRE techniques

MRI systems vary across vendors and even across models of the same vendor. Differences may include magnet design, RF coil design, gradient performance, and default sequence parameters. For instance, differences in center frequency (e.g., 123 MHz and 127 MHz in Siemens and Philips 3-T scanners, respectively) are an indication of different magnet field calibrations, which may also relate to shim calibrations. Specifically, on T1 mapping, sequence implementation varies in terms of the default pulse design used for preparation magnetization and readout, reconstruction algorithm for motion corrections, and analysis software with different fitting models. These variations produce systematic T1 value differences for the same object (Fig. 5).

The MOLLI 3(4s)3(4s)5 technique was first utilized on a Philips scanner, with investigations into the optimal acquisition parameters carried out on a 1.5-T Siemens scanner (MOLLI 3(3)3(3)5) [7, 37]. While the majority of cardiac T1 mapping studies have employed the MOLLI (e.g., schemes 3(3)3(3)5, 5(3)3, 5(3s)3) technique, a preference for specific newly developed techniques is emerging among various vendors. For instance, techniques like ShMOLLI 5(1)1(1)1 on Siemens [38] and SMART1Map on GE Healthcare [39] are gaining traction. Philips offers MOLLI 5(3s)3 and SASHA as research options. The variations in T1 mapping techniques utilized by different vendors may contribute to the discrepancies observed when comparing T1 values across vendors.

A meta-analysis by Gottbrecht et al revealed variations in T1 values between Philips and Siemens scanners, even when adjusting for field strength differences [40]. Specifically, at 1.5 T, Philips systems tend to give slightly higher T1 than Siemens, but vice versa in 3 T [40]. Differences in default FA might be one of the reasons that can best explain these differences between vendors. For instance, earlier studies on Siemens scanners using MOLLI protocols initially used a 50° FA, then changed to 35° or ShMOLLI, whereas Philips by default used MOLLI with 20° FA at 3 T [41–43]. Furthermore, differences in RF pulse design lead to differences in inversion efficiency, which is critical for minimizing T1 mapping error [44].

Scanner-specific implementations of the same sequence (e.g., MOLLI) can yield significantly different T1 values due to variations in nominal FA, TR, TE, or preparation pulses. Even within a specific vendor, different scanner models or software versions can influence T1 values. For example, Thomas et al found that T1 values measured using three different MRI scanners from the same vendors showed up to 22% difference in the apparent diagnostic threshold across scanners [45]. However, under strict standardization of the scanning protocol and the acquisition scheme implemented, it was proven that it will result in native T1 values without any significant differences across scanners [46]. Inter-scanner variability is a real concern, but with careful standardization, it can be minimized. Therefore, multicenter studies should incorporate strategies to ensure all scanners produce equivalent results. From a clinical perspective, inter-vendor variability may limit the direct application of published T1 cutoffs in multicenter studies.

**Fig. 6:**
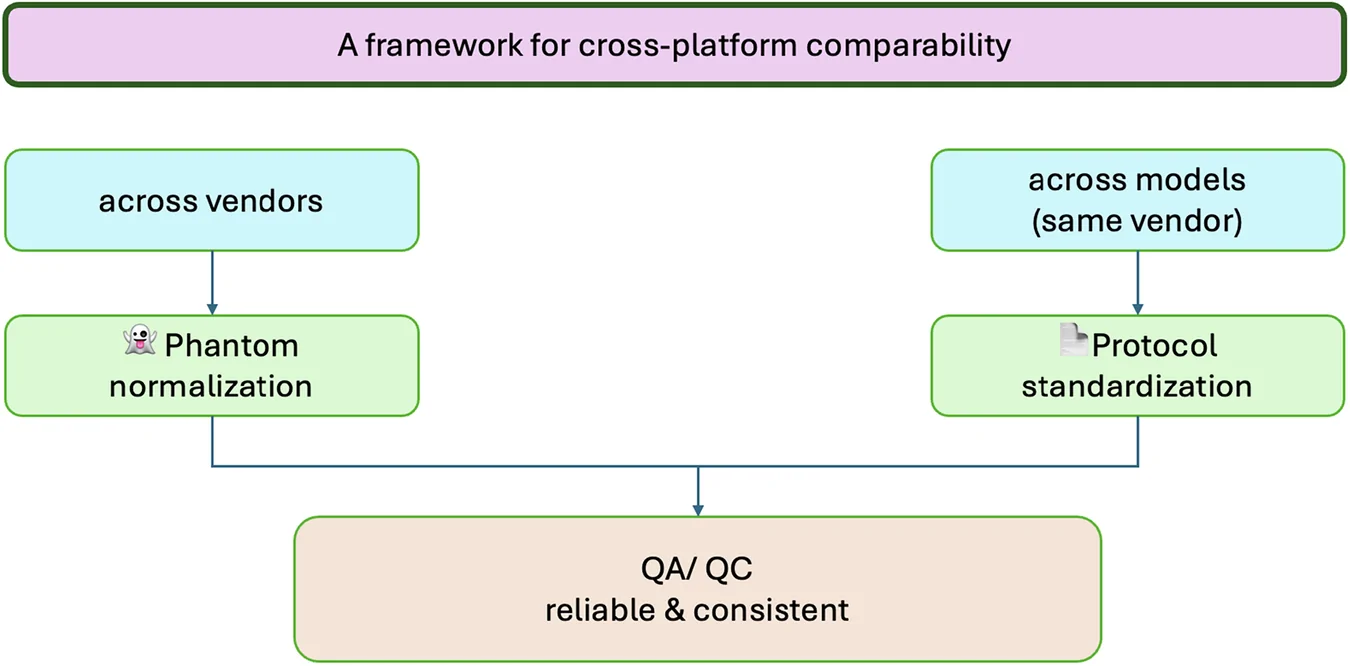
A framework for cross-platform comparability

Given the inherent variability in T1 values across vendors, field strength, and even different models from the same vendor, implementing robust quality assurance (QA) and quality control (QC) procedures becomes essential in multicenter studies to ensure reliable and consistent T1 quantification across all sites [16]. Figure 6 shows a framework for cross-platform comparability.

### Standardization and harmonization

The persistent variability in native T1 values was introduced by differences in hardware, software, and sequence implementation across MRI platforms. Even under controlled phantom conditions, considerable discrepancies have been observed. For instance, Captur et al demonstrated that identical phantoms produced significantly different T1 measurements across scanners, underscoring the influence of system-dependent factors and the need for cross-platform harmonization [47].

To address this, phantom-based correction strategies have been proposed and tested. Zhang et al recommended the development of calibration curves and scanner-specific regression models to normalize T1 values, while Suh et al confirmed that phantom correction can significantly reduce both inter-scanner and inter-subject variability [48, 49]. These findings indicate that phantom-guided harmonization is not only feasible but necessary for accurate T1 quantification across centers.

Equally important is the standardization of image post-processing. Carapella et al found that implementing structured post-processing pipelines—including consistent curve-fitting and standardized ROI placement—effectively reduced the variability [50]. They further advocate for centralized image analysis, especially in multicenter studies. Along similar lines, Popescu et al proposed the use of clustered T1 structuring to support benchmarking efforts and called for harmonization to ensure reproducibility [23].

Additionally, the Society for Cardiovascular Magnetic Resonance (SCMR) consensus recommendations emphasized phantom calibration, full disclosure of acquisition parameters, and standardized patient preparation as foundational components of reproducibility frameworks in cardiac T1 mapping [16].

## T1 mapping protocol considerations (acquisition phase and region)

The influence of T1 mapping images on results is evident, even when employing identical scanners and sequences. Differences in T1 values can result from several factors, including the timing of the scan relative to the cardiac cycle, the localization of the imaging slices, and the approach to partitioning and quantifying the cardiac muscle. These differences make it harder to set clear and consistent local reference ranges. In this section, we investigate how the cardiac phase, slice selection, and region of interest (ROI) placement can influence native T1 measurement.

### T1 image acquisition protocol: cardiac phase

**Fig. 7:**
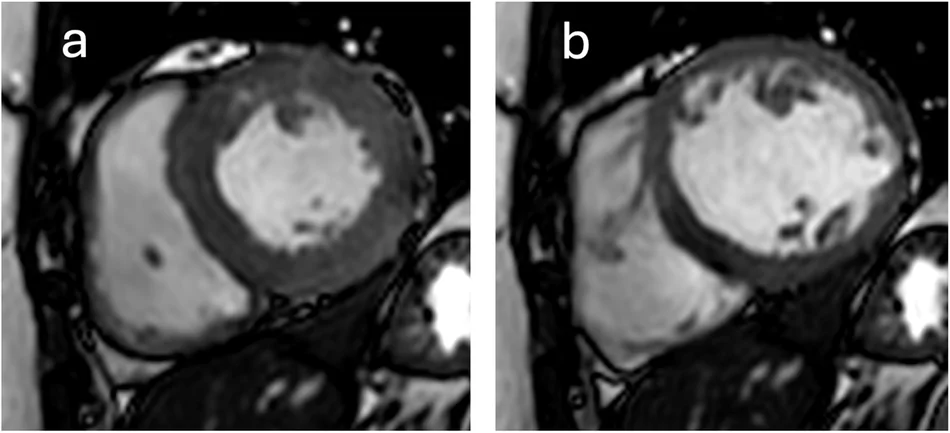
Cardiac phase during **a** end-systolic and **b** end-diastolic

When measuring myocardial T1, one must decide when and where in the cardiac cycle to acquire the images. This decision can influence the measured values and their variability. Acquisition can occur in either systole (when the heart is contracted) or diastole (when the heart is relaxed). Figure 7 shows the heart condition during systole and diastole imaged at the short axis of the left ventricle. In either case, acquisition is typically ECG-triggered such that each image in the series is acquired at a consistent time point in the cardiac cycle for all heartbeats. The majority of T1 mapping studies acquire images at mid-diastole, because this is the phase with the least cardiac motion, which can help in minimizing motion artifact [16]. However, during diastole, the myocardial wall is thinner. By contrast, in systole the myocardium is thicker (roughly double the thickness of diastole in the LV septum, for instance), which can reduce partial volume effects (mixing of myocardium and blood within a voxel) [51]. There has been interest in systolic imaging for this reason.

Empirical evidence from studies directly comparing systolic and diastolic T1 mapping within the same subjects indicates no significant variance in average myocardial T1 values between the two methods [51, 52]. Both approaches may yield comparable results, allowing researchers and clinicians to choose the timing based on factors like patient comfort or specific clinical scenarios. The choice of cardiac phase may also depend on the imaging technique used, as certain methods may better capture myocardial tissue characteristics during different phases of the cardiac cycle. Ultimately, the decision should weigh motion artifacts, myocardial thickness, and imaging study goals to ensure an accurate myocardial health assessment. Specifically, when comparing systolic and diastolic T1 values in each segment, certain segments (such as basal anteroseptal, all middle segments except inferior, and all apical segments) exhibit significantly lower systolic T1 values compared to diastolic values [53]. However, one advantage observed with systolic imaging is that it improved image quality and consistency (lower variance) in some cases because the wall is thicker and fills more of the voxel, which can help in improving the accuracy of T1 measurement [51, 54]. A study by Liu et al noted that systolic T1 mapping yielded slightly more consistent results and was less prone to gating artifacts, thus it might be preferable, especially in circumstances where the myocardium is thin (e.g., in healthy women or in diseases with thinning) [52, 55].

Current expert consensus still recommends diastolic acquisition for patients in normal sinus rhythm because of the motion artifacts [16]. It is easier to get artifact-free images in diastole. But for patients with high heart rates or arrhythmias, diastole can be very short and inconsistent. In such cases, acquiring in systole can reduce gating errors and yield better image quality. Beyond that, heart rate (HR) directly influences the accuracy of T1 estimation, particularly for inversion recovery (IR)-based sequences like MOLLI [56]. In conventional MOLLI protocols, the recovery periods between inversion cycles are fundamentally dictated by the length of the patient’s R-R intervals [7, 15]. At elevated HR (e.g., tachycardia), R-R intervals fail to provide sufficient time for the complete recovery of longitudinal magnetization between successive Look-Locker inversion cycles, leading to an increasing and progressive bias [14, 57, 58]. This incomplete recovery results in a systematic underestimation of T1 values, a phenomenon that is particularly pronounced when measuring tissue with long T1 relaxation times, such as native myocardium and blood pool [14, 59]. Clinically, this HR-dependent bias can either mimic pathology or mask true diffuse fibrosis, potentially affecting diagnosis or treatment decisions [60]. To ensure technical completeness in tachycardiac or arrhythmic patients, literature recommends utilizing saturation-recovery (SR) sequences such as SASHA [61]. Unlike IR methods, SR techniques apply a saturation pulse that nulls the longitudinal magnetization entirely before each acquisition [62]. Because the recovery curve always resets completely regardless of the cardiac cycle’s length, SASHA measurement is strictly independent of the R-R interval and immune to the HR-driven underestimation biases that affect MOLLI [14, 15].

Shinbo et al demonstrated that in atrial fibrillation or very fast heart rates, systolic acquisition improved measurement accuracy by avoiding the variable diastolic duration [54, 63]. Therefore, protocol recommendations can be patient-specific: diastole for a calm or regular heart; systole for a fast or irregular heart. There is an interesting nuance: females often have slightly higher native T1 values than males [45, 64]. One theory for this is the smaller LV mass and thinner walls in females, which could lead to more partial volume with the blood pool. Thus, systolic acquisition when the myocardium wall thickens may mitigate this. Indeed, it was suggested to perform T1 mapping during systole for women to reduce partial volume. Consequently, Ferreira et al developed a systolic ShMOLLI variant to address exactly this issue. Their work indicated more robust results in women and tachyarrhythmias when imaging during systole [65].

**Fig. 8:**
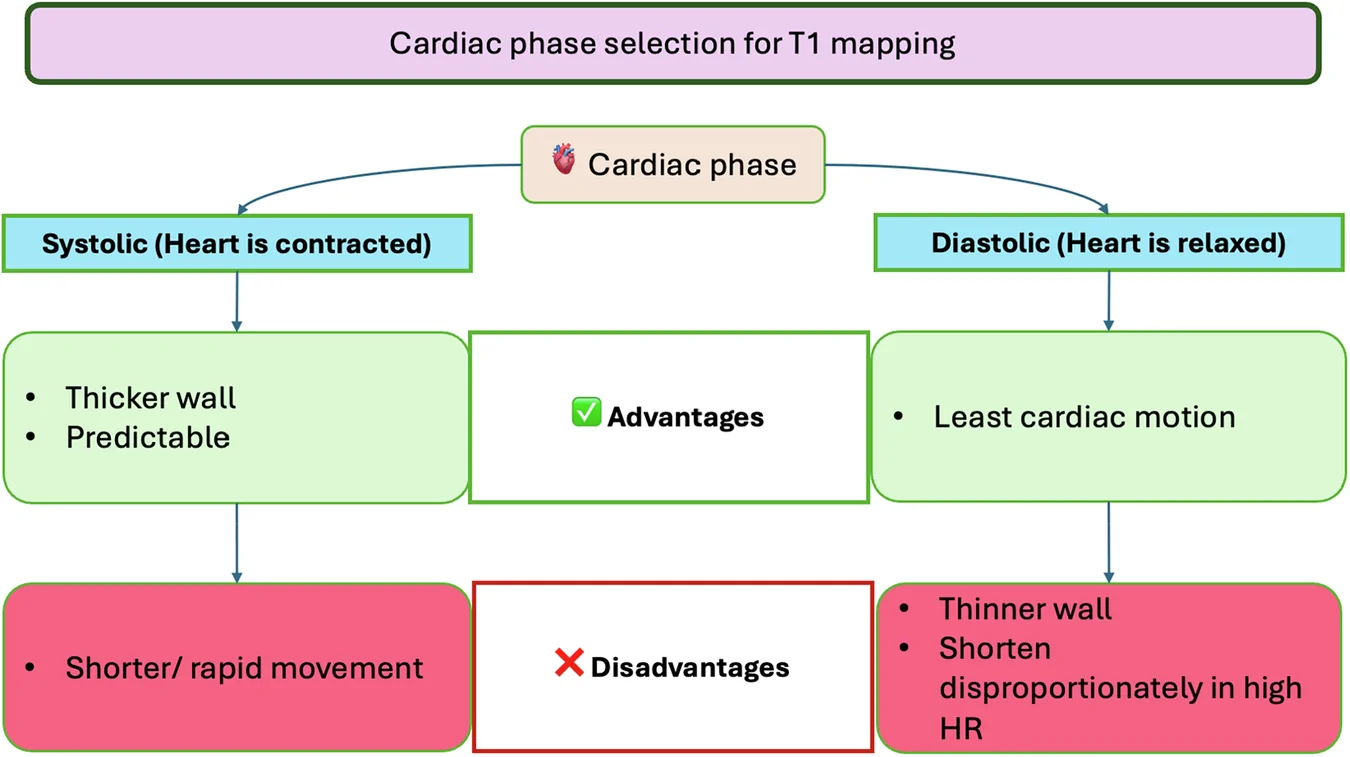
Cardiac phase selection for T1 mapping

The selected cardiac phase (systolic or diastolic) introduces physiological and acquisition-related biases. This factor is largely universal, but its effect may be amplified in certain sequences with longer breath-holds or higher sensitivity to motion (e.g., MOLLI 3(3)3(3)5). Clinically, this emphasizes the need for protocol consistency, particularly when using serial imaging to assess disease progression or treatment response. Consistency in phase selection is essential when determining a reference range. Although most normal reference range data are collected in diastole, sites that prefer systolic mapping must establish their own norms using systolic acquisition. While some studies indicate minor average differences, it is important to exercise caution as significant variations have been reported in certain segments. A summary of cardiac phase selection for T1 mapping is shown in Fig. 8.

### Slice selection, myocardial segmentation, and ROI placement

The American Heart Association (AHA) 17-segment model divides the left ventricle into basal, mid, and apical levels, providing a clear framework for placing ROIs and measuring regional T1 values consistently [66]. Building from this standardized segmentation, many studies streamline their approach by focusing on a single mid-ventricular short-axis slice, which captures key segments while maintaining diagnostic relevance [42, 67–69]. This approach is efficient and, because the mid-ventricle is often where diffuse processes manifest uniformly, it is considered representative.

Alternatively, other studies opt for multiple short-axis slices, encompassing three short-axis slices (basal, mid, and apical), each in the same positions as cine imaging, to cover 16 of the 17 standard segments (the very apex, segment 17, is often excluded because it is too thin to measure reliably) [53, 70–72]. In a few cases, researchers have extended coverage further by incorporating long-axis views, such as 4-chamber T1 maps (to ensure no segment is missed) [73]. However, such comprehensive protocols are generally reserved for research purposes, as they add complexity and are not widely adopted in routine clinical or reference mapping settings [74]. A recent study involving 78 CMR in 26 healthy volunteers demonstrated that while native T1 values in both axes are highly reproducible across scanners, significant segment-to-segment variation exists within the same axis, and up to 33% of corresponding segments between the two axes differed [73]. This finding highlights the importance of measuring more than one segment and using identical segments in follow-up studies to avoid systematic bias.

There are three common approaches to reporting T1 values, irrespective of the number of acquired slices: Global mean T1, Segmental or regional T1, and Septal T1. Global mean T1 represents the average T1 of the myocardium, excluding the apical cap, offering a single representative value. Segmental or regional T1 provides values for each standardized segment or slice level (base, mid, apex) to examine regional variation, with particular attention often given to the interventricular septum. Many studies report a “Septal T1” value, which is typically the average of the anteroseptal and inferoseptal segments at mid-ventricle (and sometimes also basal septum) [75, 76]. The reason for choosing the septal region is that it is less affected by susceptibility artifacts and B0/B1 inhomogeneities due to its anatomical location far from the lungs. Thus, it is recommended that T1 be measured in the interventricular septum as it is more shielded from artifacts and yields more consistent and highly reproducible T1 values, making it the preferred region for assessing diffuse fibrosis [76]. Table 1 presents a comparative analysis of different reported native T1 values, advantages, disadvantages, and their usage.

**Table 1:** Comparative analysis of different reported native T1 values

Metric	Global T1	Segmental T1	Septal T1
Advantages	-Reproducible -Fast acquisition	-Regional pathology -Focal lesion detection	-More consistent -Less affected by artifact
Disadvantages	-Missing heterogeneity	-Motion-sensitive	-Limited coverage
Usage	-Screening	-Ischemia/fibrosis ✅ Preferable for reporting reference range	-Diffuse disease ✅ Longitudinal/follow-up study

It has been observed that regional variation exists in T1 values across segments [77]. Some studies found T1 values in the septum might differ slightly from those in the lateral wall or that apical segments had slightly different T1 values than basal segments [53, 74, 78]. For example, a couple of studies noted a trend of T1 being a bit higher in the apex than in the base (perhaps due to partial volume with the thinner apical wall or B1 field effects). Granitz et al reported significant regional T1 differences at 3 T (lateral wall higher than septal), but not at 1.5 T [32]. As discussed earlier, technical factors like B0, B1 inhomogeneity likely explain some of these patterns at 3 T. This is supported by a finding from Kellman et al, who demonstrated that what appears as a “regional variation” in T1 (e.g., higher T1 in the lateral wall) can be largely an artifact from incomplete field shimming rather than a true physiological difference [25].

This step introduces operator-dependent and partly universal variability, particularly in the context of tissue heterogeneity, partial volume, and inclusion of adjacent structures. Inaccurate ROI placement can bias T1 measurements, especially in regions adjacent to infarction or fibrotic zones. Therefore, standardizing segmentation guidelines is essential to reduce inter-observer variability and improve reproducibility [50, 75].

Segmentation can be performed either manually or semi-automatically. Differences in operator experience, ROI size, and anatomical landmark selection can lead to considerable discrepancies in native T1 estimates, especially in regions affected by partial volume effects or artifacts. Studies have shown that inconsistent delineation of myocardial borders, such as the interventricular septum, can contribute to intra- and inter-observer variability even when acquisition protocols are standardized [13, 43, 45]. Therefore, the lack of a harmonized segmentation protocol poses a substantial barrier to reference value consistency across centers.

### Post-processing and analysis tools

Fitting models used for T1 relaxation curve reconstruction also influence results. Techniques range from simplified three-parameter exponential models to more sophisticated approaches incorporating Look-Locker correction or Bloch simulation-based estimation. For example, Roujol et al demonstrated that signal modeling differences among MOLLI, ShMOLLI, SASHA, and SAPPHIRE sequences result in varying accuracy and precision [59]. Similarly, Shao et al showed that the BLESSPC algorithm, which integrates slice profile corrections, improved accuracy by mitigating systematic bias from imperfect RF pulses [27]. Without standardization of fitting methodology, even identical raw data may yield divergent T1 values. Therefore, standardizing or at least reporting the fitting methodology is essential for meaningful comparison of results between centers and studies.

Beyond the fitting model itself, the choice of analysis platforms further complicates inter-site comparisons. In broad terms, three types of tools can be distinguished: (1) vendor-integrated clinical workstations, (2) third-party commercial packages, and (3) research-based or open-source toolboxes. These platforms differ in their default image registration strategies, motion correction algorithms, region of interest tools, and curve-fitting implementations. These can introduce center-specific bias in reported T1 values even when the acquisition protocol is similar. Notably, Popescu et al proposed structured benchmarking using clustered distributions, while Viezzer et al validated a post hoc standardization approach using z-score normalization to improve cross-platform agreement [23, 79]. In addition, Suh et al confirmed that integrating phantom-based correction into the analysis workflow could reduce both inter-scanner and inter-subject variability, emphasizing the value of unified post-processing pipelines. Taken together, these findings highlight the necessity for robust standardization not only during image acquisition but also throughout the entire post-processing chain. Harmonizing segmentation procedures, adopting validated curve-fitting approaches, and unifying analysis platforms are crucial steps toward the generation of reliable local native T1 reference ranges.

## Pulse sequence variants and mapping techniques

Various pulse sequence variants have been developed for myocardial T1 mapping in CMR, each with unique preparation schemes and acquisition strategies that can significantly influence the measured T1 values. Among these, the MOLLI sequence is the most widely used. However, several alternative designs exist. Differences in pulse sequence design can result in systematic differences in reported T1 values, complicating direct comparison across studies and sites.

Commonly used T1 mapping techniques include MOLLI, shortened MOLLI (ShMOLLI), saturation method using adaptive recovery times for cardiac T1 mapping (SMART1Map), saturation recovery single shot acquisition (SASHA), and saturation pulse prepared heart rate independent inversion recovery (SAPPHIRE) [7, 39, 61, 80, 81]. While MOLLI and ShMOLLI utilize inversion recovery (IR) for magnetization preparation, the others are based on saturation recovery (SR) methods. These differences affect the signal evolution and the fitting curve. Systematic comparison among these sequences revealed that MOLLI is the most precise, while SASHA is the most accurate, with SAPPHIRE and ShMOLLI demonstrating intermediate characteristics [59]. Both MOLLI (e.g., 5(3)3, 4(1)3(1)2) and ShMOLLI (e.g., 5(1)1(1)1) have been shown to systematically underestimate in vivo T1 values as compared to SASHA [38]. One contributing factor to the underestimation of T1 values in IR-based techniques is the cumulative magnetization transfer (MT) effects from cumulative readout and imperfect inversion, which together bias the apparent longitudinal relaxation and lead to systematically lower T1 measurements [44, 82, 83].

MT refers to the exchange in magnetization between two pools of protons (free vs bound pool). These interactions, catalyzed by repetitive RF pulses (e.g., MOLLI 5(3)3), cause partial saturation, resulting in reduced observable signal in the free pool (e.g., mobile water proton) because of the skewed recovery curve [16, 84]. Unlike MOLLI, SASHA, which is an SR-based technique, has fewer cumulative RF pulses, thereby relieving the effect of cumulative MT, making it more robust toward MT effects [85].

Furthermore, ShMOLLI typically yields T1 values about 20–25 ms lower than MOLLI, with the difference being consistent across 1.5 T and 3 T field strengths [40]. These biases are inherent to the design of the preparation pulses and readout strategy, rather than the scanner hardware, i.e., sequence-dependent bias. Table 2 presents a comparison summary among the most reported T1 mapping techniques in the literature.

**Table 2:** Comparison of different T1 mapping techniques focusing on their key features, advantages, disadvantages, and known bias

Sequence	Key features	Advantages	Disadvantages	Known biases	References
MOLLI	IR-based; original scheme 3(3)3(3)5, newer scheme shorter (e.g., 5(3)3)	High precision, widely available, high SNR, good spatial resolution, now possible with faster breath-hold, widely available on vendor platforms.	T1 underestimation, especially at high HR, imperfect inversion despite faster acquisitions	Susceptible to MT effects, inversion efficiency, HR-dependence	[7, 37, 59]
ShMOLLI	Compressed version of the original MOLLI scheme;	Originally offered a faster scan than older MOLLI; more robust at high HR; minimal user adjustment needed.	Slightly longer than optimized MOLLI (e.g., 5(3)3); lower precision than MOLLI	Still affected by MT and inversion efficiency, though less than MOLLI	[59]
SASHA	SR-based	More accurate (closer to true T1); less sensitive to MT; HR independent.	Lower SNR, more noise; longer acquisition time	Less affected by MT or inversion efficiency; but noisier signal can reduce precision	[59, 61]
SMART1Map	SR-based with adaptive delay times	Accurate T1 estimation; robust to HR and MT; minimal bias.	Less adopted; less validated in large clinical cohorts	Minimal bias; low sensitivity to MT; accurate true T1	[39]
SAPPHIRE	Hybrid method: SR and IR	Balanced accuracy and precision; designed to reduce MT effects.	Intermediate complexity; fewer validation studies	Reduced MT effects; promising accuracy-precision compromise	[59]

*MOLLI* modified Look-Locker inversion recovery, *shMOLLI* shortened MOLLI, *SASHA* saturation recovery single shot acquisition, *SMART1Map* saturation method using adaptive recovery time for cardiac T1 mapping, *SAPPHIRE* saturation pulse prepared heart rate independent inversion recovery, *IR* inversion recovery, *SR* saturation recovery, *MT* magnetization transfer

Systematic differences among techniques are further influenced by vendor-specific implementations (see “Manufacturer and scanner model variability” section). As a result, combining T1 mapping across different sequences or scanner platforms requires careful standardization. Several strategies have been proposed, including z-score normalization, post hoc correction, and clustered benchmarking [23, 79, 86]. For instance, expressing a patient’s T1 value as a z-score relative to the healthy reference specific to the sequence and scanner allows cross-scanner comparability [86]. Alternatively, Viezzer et al used a regression-based harmonization approach to reduce scanner-related bias across study sites [79], while Popescu et al introduced a vendor-independent approach using clustered benchmarking to support smaller centers in establishing local reference ranges [23].

In conclusion, while multiple T1 mapping sequences are available, each with a distinct pulse preparation scheme and technical characteristics, they result in systematic variations in reported T1 values. MOLLI continues to serve as the most used and precise technique, and SASHA is recognized for its accuracy.

## Acquisition parameters and optimization

**Fig. 9:**
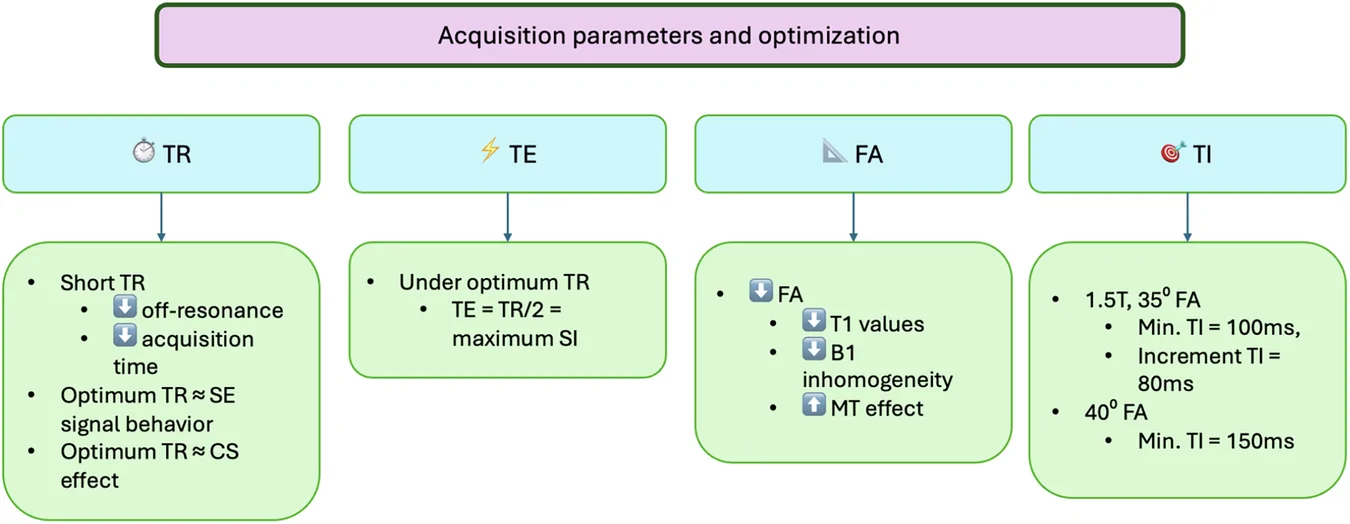
Specific acquisition parameters for bSSFP-based MOLLI and optimization

The accuracy and reproducibility of T1 mapping in CMR depend not only on the imaging sequence chosen but also on the precise configuration of its acquisition parameters. The MOLLI sequence is widely used in routine clinical practice because it provides the highest precision and high image quality. However, the ability of MOLLI to quantify native myocardial T1 is significantly influenced by various acquisition parameters, such as repetition time (TR), echo time (TE), flip angle (FA), inversion time (TI) acquisition scheme, and readout method. From a clinical perspective, inappropriate or inconsistent parameter choices can lead to significant variability in measured T1 values, undermining efforts to establish reliable local reference ranges. Therefore, practical knowledge of how TR, TE, and FA influence bSSFP-MOLLI is essential for centers aiming to implement T1 mapping as a reliable clinical biomarker. Figure 9 illustrate cquisition parameters for bSSFP-based MOLLI and optimization.

Discussion on the effect of TR on measured T1 values is relatively limited, but shortening the TR in bSSFP primarily reduces sensitivity to off-resonance effects rather than simply shortening acquisition time [25, 87, 88]. Thus, the main consideration in choosing TR is to avoid off-resonance artifacts, which is particularly important at 3 T, where susceptibility-induced field inhomogeneities are more pronounced and can directly degrade image quality.

In addition to off-resonance sensitivity, TR variation also influences T1 value estimation through its effect on the underlying T2/T1 contrast of the bSSFP signal. It has been reported that this effect is more pronounced at 1.5 T than at 3 T [23], due to field-dependent differences in T2 and T1 relaxation times. At 1.5 T, small changes in TR can subtly alter the balance of T2 and T1 contributions to the signal, thereby introducing bias in the fitted T1 values, although this issue has received limited attention in the literature. Taken together, these findings highlight that TR plays dual but field-dependent roles in bSSFP-based T1 mapping: at 3 T, the dominant concern is minimizing off-resonance artifacts to maintain signal stability; while at 1.5 T, careful consideration of TR is necessary to avoid introducing T1 bias through relaxation-related signal changes.

In practice, TR implementations vary considerably across studies and vendors[48, 72, 74, 89–91]. Rather than focusing on absolute numerical ranges, it is clinically more relevant that each site selects a TR that minimizes off-resonance artifacts and field-dependent T1 bias for its specific hardware and field strength. However, other considerations, such as the influence of fat-water chemical shift on signal, need to be taken into consideration. The selection of an appropriate TR in bSSFP-based MOLLI is paramount for accurate signal interpretation, particularly in pathological conditions like fatty metaplasia in chronic myocardial infarction [92]. In prior work, the TR was set such that water and fat signals acquire opposite phase polarities under normal on-resonance conditions, leading to an apparent signal dominated by their differences rather than their sum [93]. This phenomenon carries critical implications in fatty metaplasia cases, where fat introduces a confounding fat signal that can distort T1 measurement if unaccounted for. Crucially, an optimally chosen TR will ensure reliable T1 quantification. Thus, in this context, precise selection of TR is not merely a technical consideration but a fundamental determinant of diagnostic accuracy, underscoring its essential role in robust myocardial tissue characterization.

In conventional gradient echo sequences, the TE plays a key role in image contrast when fat is present, as it governs water-fat phase differences and can lead to signal cancellation at specific TE values. In contrast, for bSSFP sequences, the same principle of phase control applies through TR rather than TE [93]. Here, TE is inherently linked to TR, where, under optimal TR conditions, the signal behaves like a spin echo signal, with maximum intensity achieved when TE ≈ 1/2TR [94]. Clinically, TE in bSSFP-MOLLI is usually chosen in relation to TR (often close to TR/2) to maximize signal intensity, while TR remains the dominant control parameter for managing water-fat interactions and avoiding banding artifacts.

Furthermore, T1 measurement using bSSFP-based MOLLI is also influenced by the readout flip angle (FA). A higher FA produces stronger magnetization saturation, leading to T1 underestimation. While early MOLLI protocols used 50° FA [7], further optimization recommended the use of 35° for 1.5 T [37], which became standard for T1 mapping using the MOLLI technique in 1.5-T systems. Increasing FA tends to decrease measured T1 values [23], but at the same time increases the magnetization transfer effect that may improve tissue differentiation [84]. FA selection is critical for minimizing B1 inhomogeneity effects (see “Transmit RF field homogeneity (B1)” section for detailed discussion) and balancing SNR in routine clinical practice.

In a 1.5-T system, Messroghli et al in 2007 suggested optimization in acquisition parameters by using a minimum TI of 100 ms with an increment of 80 ms, which was proven to reduce heart rate dependency [37]. In the literature, there are various minimum TIs implemented, ranging from 91 ms up to 225 ms [53, 78, 95, 96]. Using a small TI will result in overestimation of T1 values [97]. Therefore, Mostafa et al suggested using a TI of 150 ms to eliminate T1 estimation error [97]. But this study was a simulation using 40° FA, which is not used in any T1 mapping clinical studies. Therefore, for harmonization of T1 values across scanners, the first crucial step is to look for the optimum acquisition parameters specific to scanner hardware and software configuration in standardizing the MOLLI protocol across scanners. Although MOLLI is available across vendors, implementation differences, such as the use of specific FA, TR, TE, and post-processing, make T1 values vendor-dependent. This variability complicates cross-vendor comparison even when using nominally the same sequence—vendor-dependent bias. Table 3 shows a summary of these specific factors, categorized into either sequence-independent or sequence-associated.

**Table 3:** Summary of related technical factors, type of bias, dependency, and their typical impact on T1 values

Category	Specific factor	Type of bias	Dependency	Typical impact on T1
Sequence-independent	B0	Systemic	Universal	Increase T1 at 3 T vs 1.5 T
	B1 inhomogeneity	Systemic	Universal	Variable depending on location
	Patient-specific (HR)	Physiological	Universal	Underestimation if HR not accounted
	Flip angle (FA)	Acquisition parameter	Vendor-dependent	Non-optimal FA = underestimation
	TR/TE	Acquisition parameter	Sequence & Vendor-dependent	Affects relaxation curve sampling
Sequence-associated	IR-based (MOLLI, ShMOLLI)	Sequence-dependent	Sequence-dependent	Underestimates T1 due to MT effects
	SR-based (SASHA, SMART1Map)	Sequence-dependent	Sequence-dependent	More accurate, less precise
	Magnetization transfer (MT) effect	Sequence-dependent	Sequence-dependent	Stronger in IR-based methods
	Sampling Scheme	Sequence-dependent	Sequence-dependent	Impacts fit accuracy and curved shape
	Fitting model	Post-processing	Software/Vendor-dependent	Over- or underestimation of T1

While most clinical MOLLI implementations use a bSSFP readout, alternative implementations with spoiled gradient echo readout (GRE) have also been proposed to mitigate off-resonance effects [27]. GRE-based T1 mapping is intrinsically less sensitive to B0 inhomogeneity and does not exhibit the banding pattern characteristic of bSSFP, which can be problematic in high susceptibility areas such as around cardiac devices. Consistent with this, Garster et al (2025) compared MOLLI with bSSFP and GRE at 1.5 T in patients with pacemakers/defibrillators and in controls [98]. They found that, with GRE, the visual artifacts are fewer than with bSSFP (24% vs 45%). Nevertheless, GRE generally has lower SNR than bSSFP [99], so reduced artifacts must be weighed against lower precision and the need for sequence-specific reference values.

In summary, optimizing acquisition parameters in MOLLI T1 mapping, especially TR, TE, FA, and TI, is not a matter of convenience but a fundamental requirement for obtaining accurate and clinically meaningful T1 values. The dual influence of TR on off-resonance artifact suppression and relaxation contrast, along with its interaction with fat signals, highlights its critical role, particularly at 3 T. Similarly, TE and FA need to be carefully calibrated to maximize signal quality while minimizing bias. While some guidelines exist, variability in implementation across different vendors and field strengths persists. Therefore, future work must prioritize the development of vendor-specific and field-strength-specific optimized MOLLI protocols. Establishing these standard acquisition settings will be the first critical step toward achieving harmonized T1 reference values and enhancing the reliability of cardiac tissue characterization across imaging centers.

## Clinical implications

Native T1 mapping has emerged as a sensitive imaging biomarker for detecting diffuse myocardial abnormalities, including fibrosis, inflammation, edema, and infiltrative diseases—often before the onset of overt functional impairment [12, 14]. However, the diagnostic utility of T1 mapping is contingent upon comparison with appropriate normative data. In clinical practice, misclassification of myocardial tissue status may occur if locally derived values are not available, particularly in borderline cases or early disease, where subtle elevations in T1 may be pathognomonic.

For instance, recent work has highlighted how sex-specific differences in native T1 values—if not accounted for—can lead to misdiagnosis in female patients [45]. Likewise, large-scale meta-analyses have shown wide variability in reference ranges even among healthy populations, underscoring the need for population-specific reference ranges [40, 95]. In reality, clinicians increasingly rely on parametric mapping to make nuanced decisions about therapy escalation, surveillance strategies, or even to determine the need for invasive biopsy.

## Conclusion

Native T1 values are strongly influenced by magnetic field strength, B0/B1 inhomogeneities, and scanner differences, all of which complicate the establishment of robust reference ranges. Harmonization and standardization of T1 mapping protocols, together with the implementation of structured quality assurance programs, is essential to ensure reliable and comparable results across sites and vendors. Optimizing the MOLLI acquisition parameters, including repetition time (TR), echo time (TE), inversion time (TI), and flip angle (FA), can reduce variability and improve accuracy. While the MOLLI protocol has advanced T1 mapping, inter-study differences in protocol configuration and scanner settings continue to introduce variability. Furthermore, MRI system manufacturers should play an active role in defining system-specific reference ranges for their MRI platforms, thereby supporting clinical centers in establishing robust, site-specific baselines for healthy myocardium.

## Data Availability

The data supporting the findings of this study are available from the corresponding author upon reasonable request.

## Abbreviations

B0: Static magnetic field
B1: Transmit radiofrequency field
bSSFP: Balance steady-state free precession
CMR: Cardiac magnetic resonance imaging
FA: Flip angle
LV: Left ventricle
MOLLI: Modified Look-Locker inversion recovery
MRI: Magnetic resonance imaging
QA: Quality assurance
QC: Quality control
RF: Radiofrequency
ROI: Region of interest
SAPPHIRE: Saturation pulse prepared heart rate independent inversion recovery
SASHA: Saturation recovery single shot acquisition
ShMOLLI: Shortened Modified Look-Locker inversion recovery
SMART1Map: Saturation method using adaptive recovery times for cardiac T1 mapping
T1: Longitudinal relaxation time
T2: Transverse relaxation time
TE: Echo time
TI: Inversion time
TR: Repetition time
